# Supplementary material for: Southward expanding plate coupling due to variation in sediment subduction as a cause of Andean growth
Source: Nat Commun. 2021 Dec 14;12:7271. doi: 10.1038/s41467-021-27518-8 (PMC8671423; doi:10.1038/s41467-021-27518-8)
Supplement: Supplementary file 1 — Supplementary Information [file 41467_2021_27518_MOESM1_ESM.pdf]

Supplementary information of ”Southward expanding plate coupling due to climate-tectonic interaction as a cause of Andean growth”

Jiashun Hu[1,2,3*], Lijun Liu[3] & Michael Gurnis[2]

[1]*Department of Earth and Space Sciences, Southern University of Science and Technology, Shenzhen, China 518055*

[2]*Seismological Laboratory, California Institute of Technology, Pasadena, CA, USA 91125*

[3]*Department of Geology, University of Illinois at Urbana-Champaign, Champaign, IL, USA 61801*

*corresponding author: hujs@sustech.edu.cn.

Content:

Supplementary Discussion

Supplementary Figures 1-11

Supplementary Tables 1-2

Supplementary References

Supplementary Discussion

Trench-axial sediment transport The trench-fill sediment distribution is controlled by sediment supply from either the continental margin or the ocean basin, sediment consumption by either subduction to the mantle or accretion to the overriding plate, and sediment transport within the trench. It has been proposed that latitudinal sediment transport within the trench played an important role for the redistribution of sediments in the trench along the Chilean coast[1–3]. First, reflection seismic profiles have revealed a prominent trench axial channel that is continuous from 44°S to 31°S[3]. This channel is 5 km wide and up to 150 m deep, which is actively transporting sediments through turbidity currents, because otherwise the channel would have been burried within 300 kyr assuming a sedimentation rate of 50 cm/ka[3]. Second, according to the computation of trench sediment volume by Volker et al.[3] based on 30 reflection seismic profiles, the sediment supply south of 43° is relatively high compared to other segments along the Chilean coast because of high glacial erosion, but the sediment volume stored in the trench along this segment is relatively low. Similarly, local maxima in trench sediment volume do not appear near the major submarine canyon outlets along the Chilean coast[3]. Both suggest a massive latitudinal transport of sediments within the trench[3]. Third, the asymmetry of the large fan systems at the exit of submarine canyons with deposition in the south but lag deposits and erosional features in the north, indicates the northward preferred diretion of turbidity currents entering the trench[4,5]. Fourth, the broad flat sedimentary plain to the south of Juan Fernandez Ridge (JFR), in contrast to the sediment starvation to the north, also suggests a general northward sediment transport which is blocked by the JFR. Finally, sediment transport along the trench through trench-axial channels has also been documented at other subduction zones, such as in the eastern Aleutian trench[6,7].

Along the Chilean coast, the trench depth at the deformation front increases from 3200 m at 46°S to 5950 m at 32°, thus the gradient of the gravitional potential energy along the northward inclining topography could be an important driving force for the northward sediment transport[3]. We suggest this topography pattern has remained stable since the onset of Andean shortening in Eocene. The topography is determined by both the depth of the oceanic basement and the thickness of sediments. The younger oceanic plate and higher sediment supply both contribute to the shallower trench depth in southern Chile compared to the northern Chile. Since the Eocene, the paleolatitude of South American Plate has remained largely stable, thus the existence of the cold upwelling Humboldt Current and the prevailing wind favour a long-term climate pattern where the northern Chile is more arid than the southern Chile, thus more sediment supply in the south than the north. And due to the subduction of the Chile Rise in southern Chile, the age of the oceanic plate has always been younger than that in the northern Chile[8], thus favouring a shallower oceanic basement in southern Chile. Therefore, we suggest the northward trench-axial sediment transport has persisted throughout the history of Andean shortening.

Sediment transport blocked by JFR Several earlier studies have suggested that the northward trench-axial sediment transport within the trench of Chile is blocked by the JFR[2,3,9]. The slope of the sedimentary basin south of the JFR is relatively gentle compared to the sharp slope of the oceanic basement induced by the seamounts whose height could reach a few kilometers. Therefore, the turbidity current could easily lose its momentum when climbing the JFR seamounts, and thus could not transport sediment further north. This is evidenced by the absence of thick sheet tuibidite deposits in the north of the ridge, resulting in a sudden drop in sediment thickness from 2.3 km north of the ridge to <0.5 km south of the ridge (Fig. 1c). in contrast, the sedimentary basin south of the JFR is thick and flat (Supplementary Fig. 1), suggesting the accumulation of sediments after being transported to the south of JFR[9].

Similar scenarios have also been observed along the Hikurangi Trough[10] and the eastern Aleutian Trench[6]. At the northern end of the Hikurangi Trough, the broad flat plain of the trough filled with sediments changes abruptly to the narrow, V-shaped southern Kermadec trench deprived of turbidites. This is because a chain of trench-intersecting low seamounts blocked the northward passage of distal turbidity currents[10]. Note in this case, thicker sediments in the Hikurangi Trough do not necessarily cause less shortening in the overriding plate. This is because the collision between the Hikurangi oceanic plateau and the Australian Plate has greatly increased the stress in the overrding plate. Along the eastern Aleutian Trench, the sediment pattern is more complex, but it is evident that the seamount chains could dam the turbidity currents, dividing the sediment system into discrete patterns[6].

Sediment subduction and plate coupling As indicated in the main text, theoretical analysis and geophysical, geological and experimental observations have suggested that subduction of well-stratified fine-graindded sediments could weaken or lubricate the plate interface through several mechanisms, including the low frictional resistance of sediments[11], elevated pore pressure by releasing fuilds from the sediments[12], lower viscosity of the sediments compared to the subducted mafic rocks[13] and thermal blanketing of oceanic basement by sediments[14]. Statistical analysis provides further evidence for the association of weak plate coupling with thick sediments. Based on a compilation in 2015, historical great megathrust earthquakes tend to nucleate at thick-sediment trenches, with 53% $\geq$Mw8.0, 75% $\geq$Mw8.5 and 100% $\geq$Mw9.1 megathrust earthquakes associated with thick sediments ($>$1km), despite the shorter global length of thick-sediment trenches[15]. This can be best explained by the weakening of megathrust faults by sediment subduction which allows the earthquake rupture to propagate over a wider area. By analyzing the force balance between the buoyancy stress due to the high Andes and forces at plate interface, Lamb and Davis[14] has computed the average shear stress at plate interface that

varies from 35–50 MPa in central Andes to <35 MPa to the north and south. This suggests a stronger plate coupling in central Andes and a weaker plate coupling in southern and northern Andes, which correlates well with the sediment distribution with thick sediments in central Andes and thin sediments to the north and south[14].

Correlation between sediment thickness and upper plate strain As discussed in the above section, thick sediments could reduce the friction coefficient of the subduction channel and weaken the plate interface. On the other hand, numerical models have shown that a high friction coefficient or a high stress at the subduction channel results in strong crustal shortening[16] (Fig. 2 and Supplementary Figs. 4-6). Therefore, one would infer that thick sediment tends to cause crustal extension, while sediment depletion tends to cause crustal shortening. Observationally, statistical analysis on present-day subduction parameters has found a weak but non-random positive correlation between sediment thickness and upper plate strain (UPS) (positive for extension), with a Spearman correlation coefficient of 0.41[17], suggesting trenches with thick sediments tend to have extensional UPS. In fact, they noticed trenches with sediments $\geq$ 1 km thick are rarely associated with compressive UPS. The only two exceptions are the Colombia and Manila trench sections which exhibit large lateral variation in sediment thickness.

We suggest sediment thickness is an important but not the only controlling factor of UPS. The interplate stress due to the differential motion between the trench and the upper plate, and the area of the plate interface both could have played a role. For example, several sediment-starved trenches are associated with backarc extension, such as the Scotia, Tonga and Mariana. These trenches typically feature a fast trench rollback controlled by mantle dynamics[18,19] and a limited area of plate interface (that is several times smaller than that of the Andes) due to the steep subduction[18,20] and the thin overriding plate. As a result, these trenches may have a weak to-

tal interplate coupling despite the thin trench sediments. Additionally, the correlation between sediment thickness and UPS is likely non-linear due to its complex physical mechanism. A positive feedback between sediment thickness and UPS may exist in central Andes, where sediment starvation causes Andean growth through crustal shortening, and the growth of Andes in turn leads to a stronger rain-shadow effect which reduces the sediment supply and a larger pressure on the plate interface which may further reduce the amount of the entrained sediments. Therefore, The effect of sediment subduction is likely more significant in this shallow-dipping compressive subduction system within the central Andes.

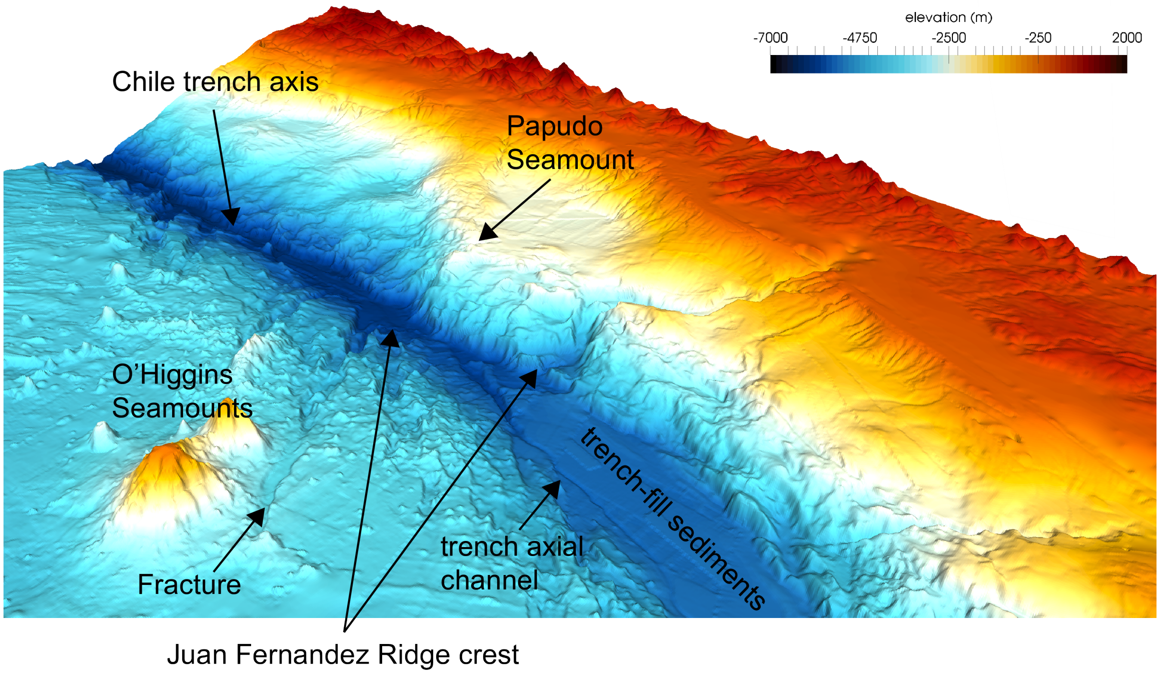

Supplementary Fig. 1. Trench-fill sediments along the coast of central Chile at about ∼35°S. 3D regional relief is presented with color indicating the elevation. The trench-fill sediments are thick south of the Juan Fernandez Ridge crest, and decreases dramatically north of the ridge.

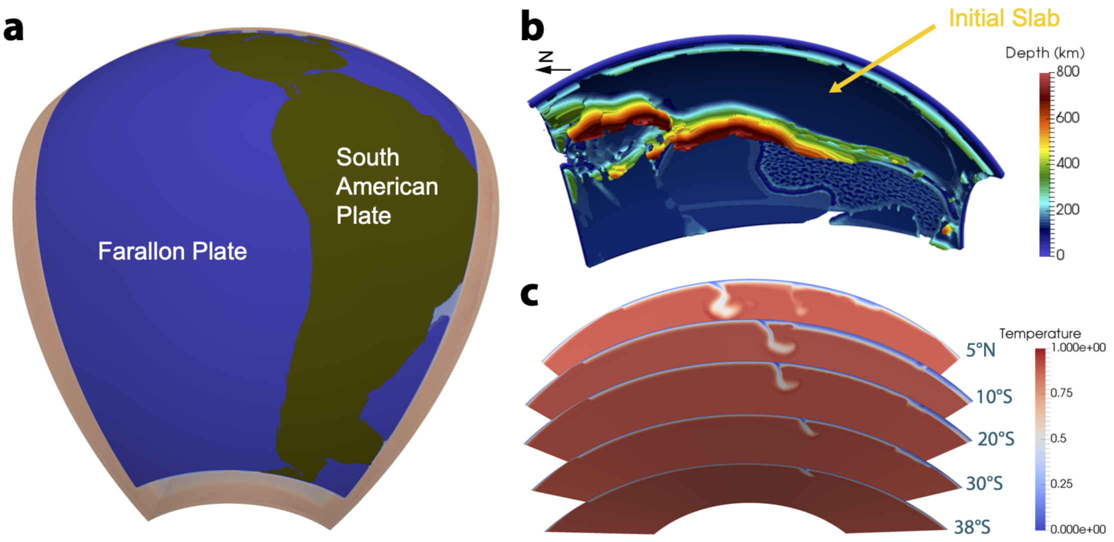

Supplementary Fig. 2. Initial condition of the numerical models. **a**, the top surface of the model domain. Continent and ocean are indicated with olive and blue. The area near the model boundaries as indicated with pink has a low viscosity to decouple the mantle flow from the boundary. **b**, initial slab structure by looking down through the mantle. Color of the slab indicates depth. **c**, initial temperature field along the cross sections at 5°N, 10°S, 20°S, 30°S and 38°S.

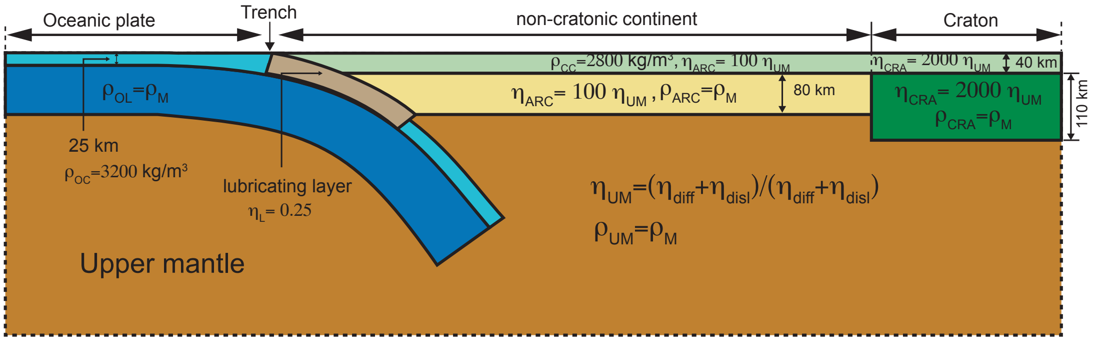

Supplementary Fig. 3. Model setup. Different compositions with different density and viscosity are shown in a cross section. The light brown area at the plate interface represents the lubricating layer. ρ and η represent the density and viscosity of the composition. The subscription M, UM, OC, CC, OL, ARC and CRA represent the mantle, the upper mantle, the oceanic crust, the continental crust, the oceanic mantle lithosphere, the non-cratonic mantle lithosphere and the cratonic mantle lithosphere, respectively.

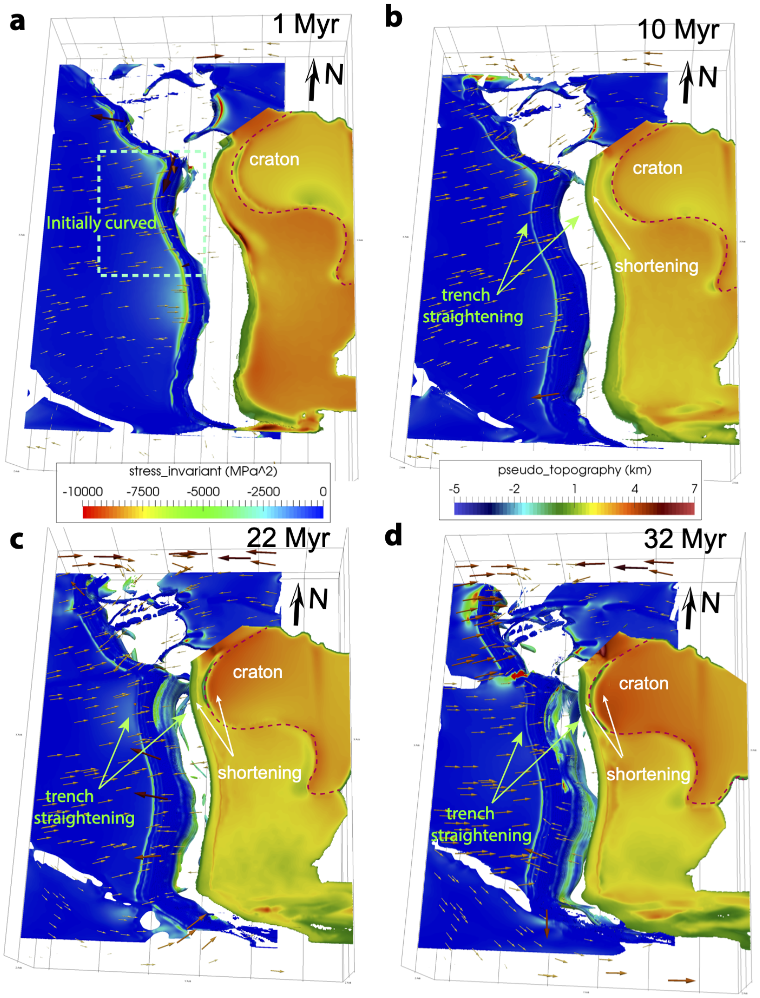

Supplementary Fig. 4. 3D representation of the evolution of model 1 (Fig. 2a). **a**, **b**, **c** and **d** show the model at 1, 10, 22 and 32 Myrs, respectively. Plates are represented with isovolume that has a non-dimensional temperature ≤ 0.7. Topography is shown on South American plate, which is used to indicate crustal shortening. The second invariant of the stress tensor is shown on the oceanic plates. The non-cratonic continent subsides before about 10 Myrs for all the three models. This is because early stage of subduction is usually associated with extension and the comparably weak rheology in non-cratonic region enlarges this effect. Other factors such as the continuous cooling of the continental lithosphere, the increasing negative dynamic topography,

and the intra-plate deformation due to the large gravitional potential energy associated with the high topography, could also contribute to the initial subsidence. We suggest these factors have limited effects on the differential shortening along the Andes. For the purpose of this study, the differential topography along the trench is the first-order topographic feature.

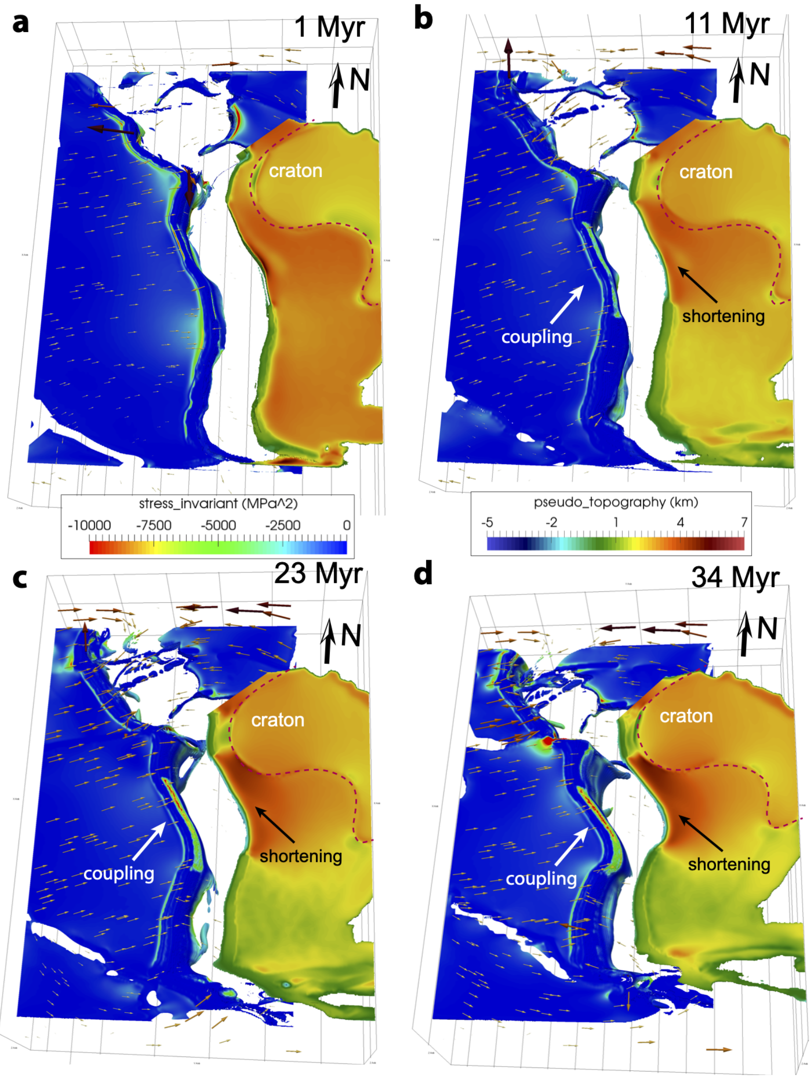

Supplementary Fig. 5. 3D representation of the evolution of model 2, where the plate coupling zone covers the region from 12°S to 33°S which does not change with time (Fig. 2b), to mimic a stationary JFR. Other settings are the same as in Supplementary Fig. 4.

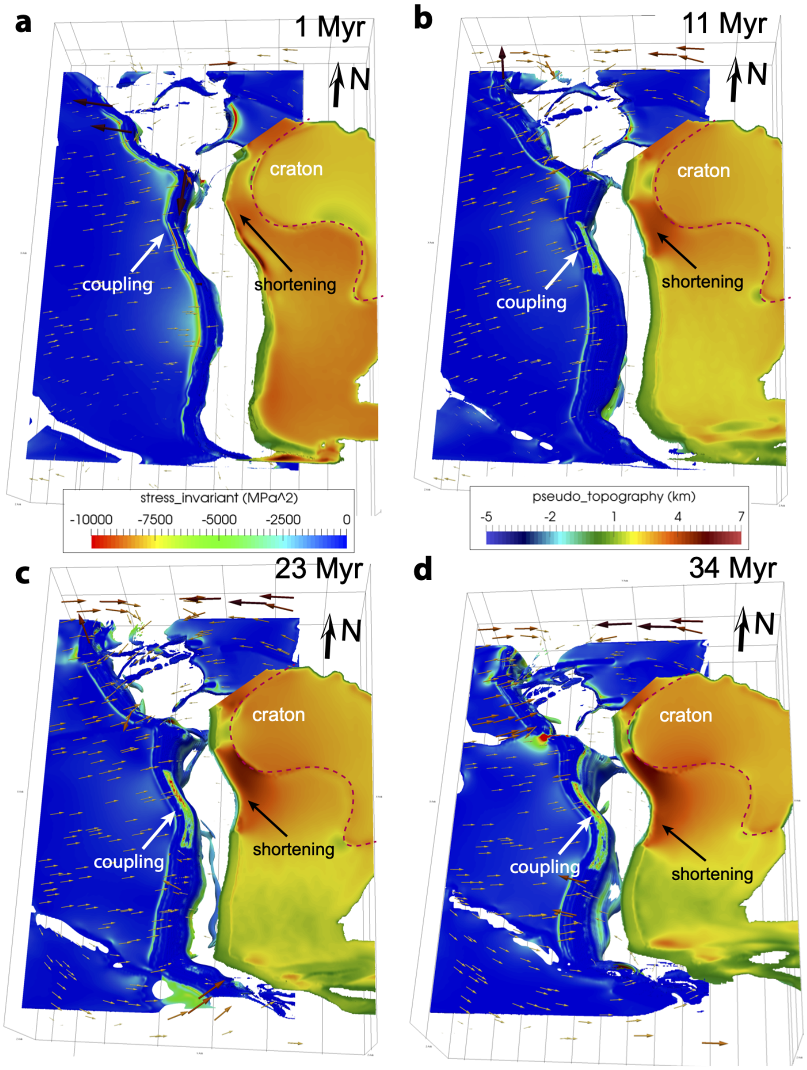

Supplementary Fig. 6. 3D representation of the evolution of model 3, where the initial plate coupling zone was narrow but expands southward progressively (Fig. 2c) to mimic the southward-migrating JFR. Other settings are the same as in Supplementary Fig. 4.

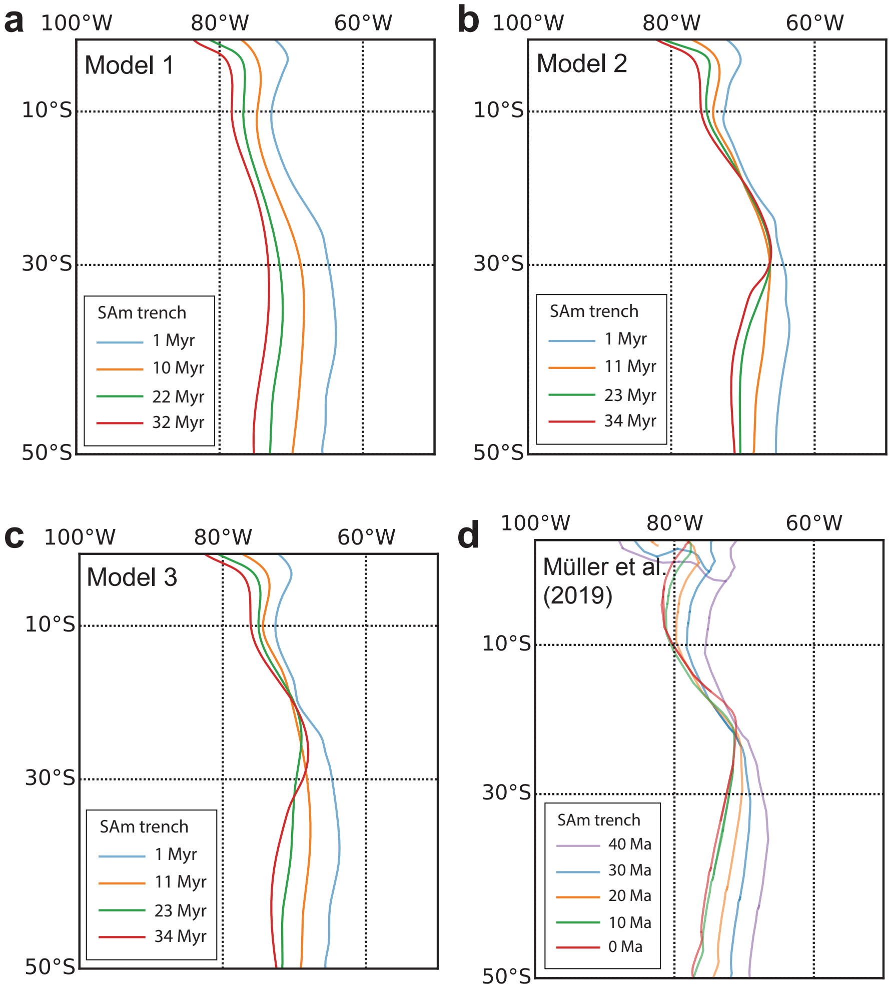

Supplementary Fig. 7. Motion of the South American trench. **a**, **b** and **c** represent the trench motion from the geodynamic models. **d** represents the trench motion from a recent plate reconstruction model[21]. Trenches at different ages are shown with different colors. Note the reconstruction model includes some northward motion of the trench which the geodynamic models do not aim to capture.

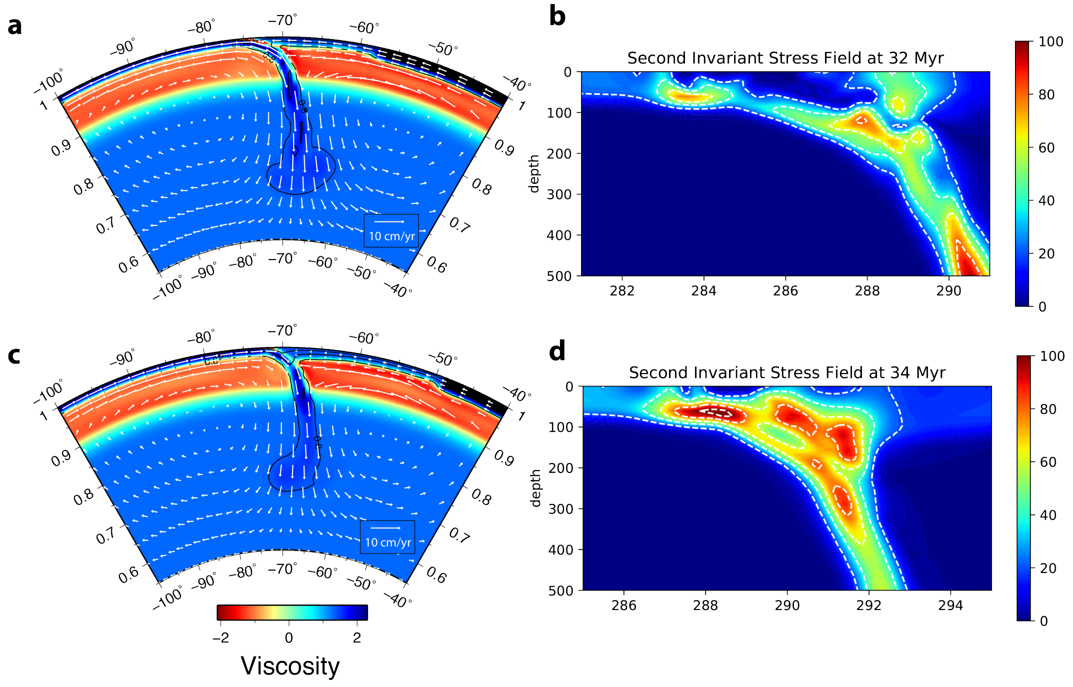

Supplementary Fig. 8. Comparison of the viscosity and second invariant stress fields between model 1 and model 3. **a** and **b** show model 1 at 32 Myrs. **c** and **d** show model 3 at 34 Myrs. Note the wider weak zone implemented in **a** (model 1), compared to **c** (model 3). **d** (model 3) has larger stress in the subduction zone than that in **b** (model 1).

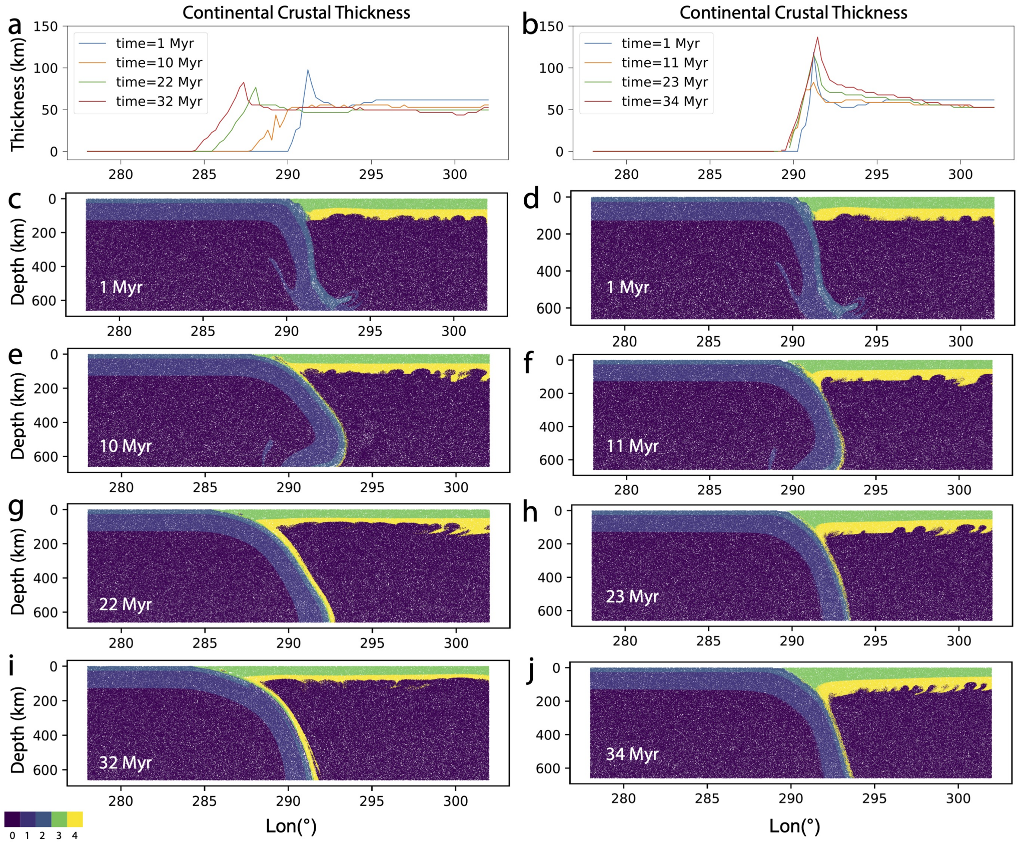

Supplementary Fig. 9. Compositional evolution of model 1 (left panel) and model 3 (right panel) at 20°. **a** and **b** show the evolution of the thickness of the continental crust of the two models. **c**, **e**, **g** and **i** show the compositional fields of model 1 at 1, 10, 22 and 32 Myr, respectively, **d**, **f**, **h** and **j** show the compositional fields of the model 2 at 1, 11, 23 and 34 Myr, respectively, Compositional labels 0, 1, 2, 3 and 4 represent the mantle, oceanic mantle lithosphere, oceanic crust, continental crust and continental mantle lithosphere, respectively. The continental lithosphere is partially destroyed by the mantle flow, because its viscosity is not very large ($2.5 \times 10^{22} Pa \cdot S$) and its density is slightly higher than the asthenosphere due to thermal effect, but it protects the crustal layer that signals the crustal shortening.

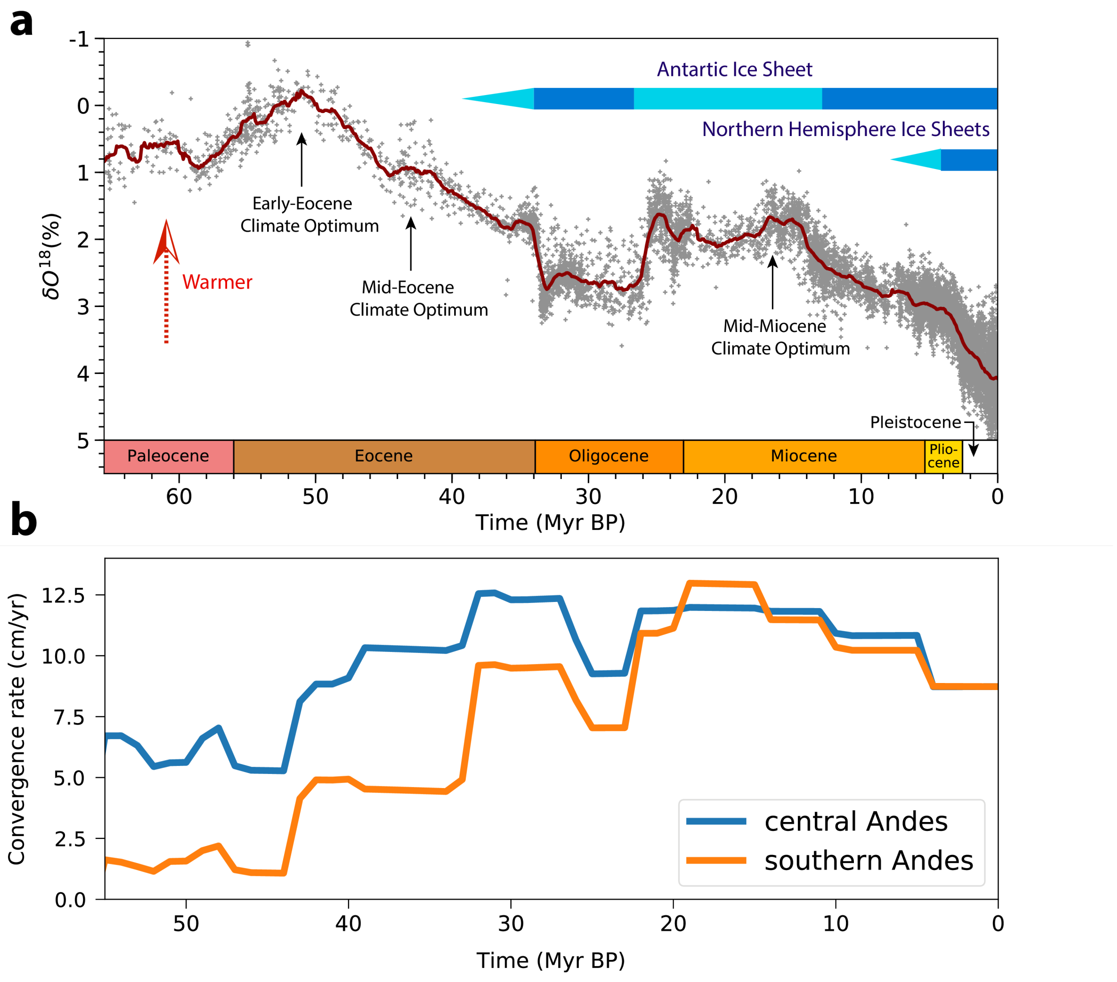

Supplementary Fig. 10. Long-term trends (**a**) in benthic oxygen isotope ratios[22] since Paleocene, which reflects the cooling of the deep ocean. "+" represents individual data points. The red line is the moving average of the data with a window size of 1 Myr. Antarctic Ice Sheet and Northern Hemisphere Ice Sheets are also indicated. **b**, convergence rate between the Nazca Plate and the South American Plate in central Andes at 20°S and southern Andes at 40°S, based on a recent plate reconstruction[8].

a

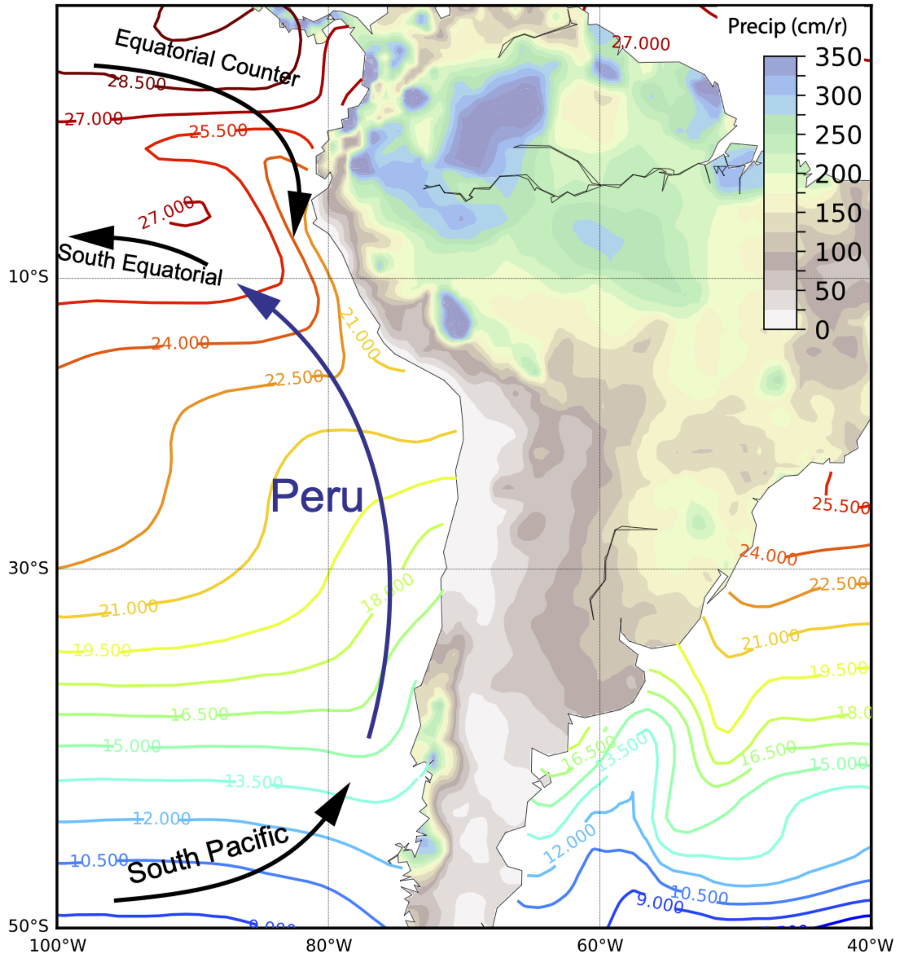

Supplementary Fig. 11. Long-term April sea surface temperature (`NOAA_OI_SST_V2` data provided by the NOAA/OAR/ESRL PSD, Boulder, Colorado, USA, from their website at https://www.esrl.noaa.gov/psd/)[23] and mean annual precipitation of South America[24]. Colored lines represent the contours of the sea surface temperature. Colored map represents the mean annual precipitation of the South American continent. Oceanic currents are indicated with blue and black arrows.

Supplementary Table 1. Basic model parameters.

Parameter	Symbol	Nondimensional value	Dimensional value	Units
Earth radius	R_0	1	6.371×10^6	m
reference density	ρ_0	1	3340	$kg \cdot m^{-3}$
thermal diffusivity	κ	-	10^{-6}	$m^2 s^{-1}$
gravitational acceleration	g	-	9.81	$m \cdot s^{-1}$
thermal expansivity	α_0	-	3.0×10^{-5}	K^{-1}
reference viscosity	η_0	1	10^{21}	$P_a \cdot s$
Rayleigh number	R_a	-	3.38×10^8	-
mantle temperature	T_m	1.0	1300	$°C$

Supplementary Table 2. Parameters for the viscosity law. Please refer to Supplementary Fig. 3 for compositional viscosity.

Variable	Upper mantle dislocation creep	Upper mantle diffusion creep	Lower mantle diffusion creep
σ_y	$200MPa$		$200MPa$
$A(r)$	5 $\times 10^{-10}$	0.25	20
n	3.5	1	1
E_a	18	18	18
T_{off}	0.1	0.1	0.6

Supplementary References

1. Von Huene, R. *et al.* Tectonic control of the subducting Juan Fernández Ridge on the Andean margin near Valparaiso, Chile. *Tectonics* **16**, 474–488 (1997).

2. Hoffmann-Rothe, A. *et al.* Oblique Convergence along the Chilean Margin : Partitioning , Margin-Parallel Faulting and Force Interaction at the Plate Interface. In *The Andes*, December, 125–146 (Springer, 2006).

3. Völker, D., Geersen, J., Contreras-Reyes, E. & Reichert, C. Sedimentary fill of the Chile Trench (32–46°S): volumetric distribution and causal factors. *Journal of the Geological Society* **170**, 723–736 (2013).

4. Thornburg, T. M. & Kulm, L. D. Sedimentation in the chile trench: Depositional morphologies, lithofacies, and stratigraphy. *Geological Society of America Bulletin* **98**, 33–52 (1987).

5. Völker, D. *et al.* Latitudinal Variation in Sedimentary Processes in the Peru-Chile Trench off Central Chile. *The Andes* 193–216 (2006).

6. Dobson, M. R. & O'Leary, D. Sediment delivery and depositional processes along the eastern aleutian trench. *Journal of the Geological Society* **176**, 1076–1092 (2019).

7. von Huene, R. Modern trench sediments. In *The geology of continental margins*, 207–211 (Springer, 1974).

8. Müller, R. D. *et al.* Ocean Basin Evolution and Global-Scale Plate Reorganization Events Since Pangea Breakup. *Annual Review of Earth and Planetary Sciences* 107–138 (2016).

9. Laursen, J. & Normark, W. R. Late quaternary evolution of the san antonio submarine canyon in the central chile forearc (33 s). *Marine Geology* **188**, 365–390 (2002).

10. Lewis, K. B., Collot, J.-Y. & Lallem, S. E. The dammed hikurangi trough: a channel-fed trench blocked by subducting seamounts and their wake avalanches (new zealand–france geodynz project). *Basin Research* **10**, 441–468 (1998).

11. Kopf, A. & Brown, K. M. Friction experiments on saturated sediments and their implications for the stress state of the nankai and barbados subduction thrusts. *Marine Geology* **202**, 193–210 (2003).

12. Bangs, N. *et al.* Broad, weak regions of the nankai megathrust and implications for shallow coseismic slip. *Earth and Planetary Science Letters* **284**, 44–49 (2009).

13. Behr, W. M. & Becker, T. W. Sediment control on subduction plate speeds. *Earth and Planetary Science Letters* **502**, 166–173 (2018).

14. Lamb, S. & Davis, P. Cenozoic climate change as a possible cause for the rise of the Andes. *Nature* **425**, 792–797 (2003).

15. Scholl, D. W. *et al.* Great ($\geq$ mw8. 0) megathrust earthquakes and the subduction of excess sediment and bathymetrically smooth seafloor. *Geosphere* **11**, 236–265 (2015).

16. Sobolev, S. V. & Babeyko, A. Y. What drives orogeny in the Andes? *Geology* **33**, 617–620 (2005).

17. Heuret, A., Conrad, C. P., Funiciello, F., Lallemand, S. & Sandri, L. Relation between subduction megathrust earthquakes , trench sediment thickness and upper plate strain. *Geophysical Journal International* **39**, 1–6 (2012).

18. Lallemand, S., Heuret, A. & Boutelier, D. On the relationships between slab dip, back-arc stress, upper plate absolute motion, and crustal nature in subduction zones. *Geochemistry, Geophysics, Geosystems* **6** (2005).

19. Stegman, D. R., Freeman, J., Schellart, W. P., Moresi, L. & May, D. Influence of trench width on subduction hinge retreat rates in 3-d models of slab rollback. *Geochemistry, Geophysics, Geosystems* **7** (2006).

20. Schellart, W. P. Overriding plate shortening and extension above subduction zones: A parametric study to explain formation of the andes mountains. *Geological Society of America Bulletin* **120**, 1441–1454 (2008).

21. Müller, R. D. *et al.* A global plate model including lithospheric deformation along major rifts and orogens since the triassic. *Tectonics* **38**, 1884–1907 (2019).

22. Zachos, J., Pagani, M., Sloan, L., Thomas, E. & Billups, K. Trends , Rhythms , and Aberrations in Global Climate 65 Ma to Present. *Science* **292**, 686–694 (2001).

23. Reynolds, R. W., Rayner, N. A., Smith, T. M., Stokes, D. C. & Wang, W. An improved in situ and satellite sst analysis for climate. *Journal of climate* **15**, 1609–1625 (2002).

24. Legates, D. R. & Willmott, C. J. Mean seasonal and spatial variability in gauge-corrected, global precipitation. *International Journal of Climatology* **10**, 111–127 (1990).
